# Atypical hemolytic uremic syndrome: a rare complication of postendoscopic retrograde cholangiopancreatography pancreatitis

**DOI:** 10.1002/jpr3.70014

**Published:** 2025-03-10

**Authors:** Apoorva Nanagiri, Samantha Pravder, Sonia Solomon, Virender Tewari, Howard Bostwick

**Affiliations:** ^1^ Department of Pediatric Gastroenterology & Nutrition, Maria Fareri Children's Hospital/Westchester Medical Center Health Network New York Medical College, & Boston Children's Health Physician Network Valhalla New York USA; ^2^ Department of Nephrology Maria Fareri Children's Hospital/Westchester Medical Center Health Network New York Medical College, & Boston Children's Health Physician Network Valhalla New York USA; ^3^ Department of Gastroenterology & Nutrition, Westchester Medical Center Health Network New York Medical College, & Boston Children's Health Physician Network Valhalla New York USA

**Keywords:** choledocholithiasis, eculizumab, renal impairment, Thrombotic microangiopathy

## Abstract

Endoscopic retrograde cholangiopancreatography (ERCP) is a crucial procedure for diagnosing and managing conditions affecting the pancreas and biliary tract. The procedure can be technically challenging and carries risks of complications, with post‐ERCP pancreatitis (PEP) being the most common. We report a case of a 16‐year‐old female who presented with cholelithiasis that progressed to choledocholithiasis which was removed using ERCP. Following this, she developed PEP and subsequently progressed to atypical hemolytic uremic syndrome, a rare complication reported in the pediatric literature.

## 1 INTRODUCTION

Endoscopic retrograde cholangiopancreatography (ERCP) has evolved to become the primary treatment for choledocholithiasis. Complications of ERCP include infection, hemorrhage, perforation, and pancreatitis. Hemolytic uremic syndrome (HUS) is a type of thrombotic microangiopathy (TMA) defined by nonimmune mediated hemolytic anemia, thrombocytopenia, and renal dysfunction. We describe a rare case in the pediatric literature of the development of aHUS as a complication of post‐ERCP pancreatitis (PEP).

## 2 CASE REPORT

A 16‐year‐old female, 3 months postpartum, presented to the emergency room with worsening right upper quadrant abdominal pain which began during her third trimester. Laboratory evaluation showed normal complete blood count, electrolytes, Creatinine (Cr), lipase, coagulation profile and bilirubin and elevated aspartate aminotransferase (AST = 107 U/L, normal <25 U/L), alanine aminotransferase (ALT = 154 U/L, normal <30 U/L), and gamma‐glutamyl transferase (GGT = 181 U/L, normal <26 U/L). An abdominal ultrasound revealed cholelithiasis without cholecystitis, and the common bile duct (CBD) measured 5 mm.

**Figure 1:**
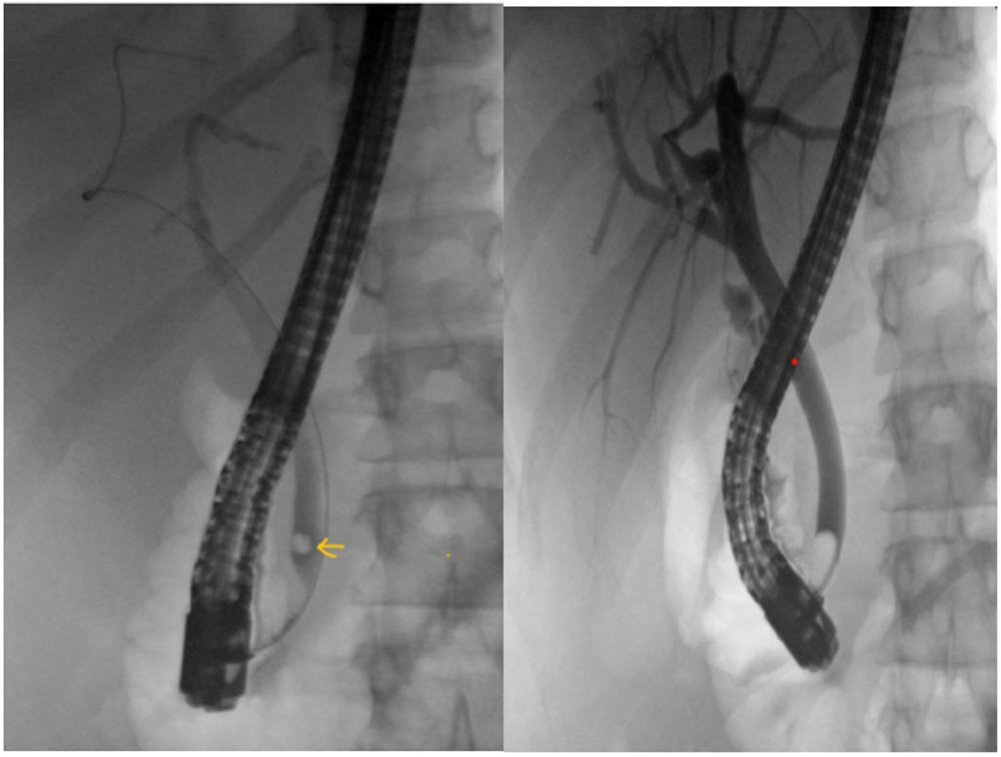
Left: Fluoroscopic image showing choledocholithiasis (arrow). Right: Occlusion cholangiogram demonstrating bile duct clearance after stone extraction.

**Figure 2:**
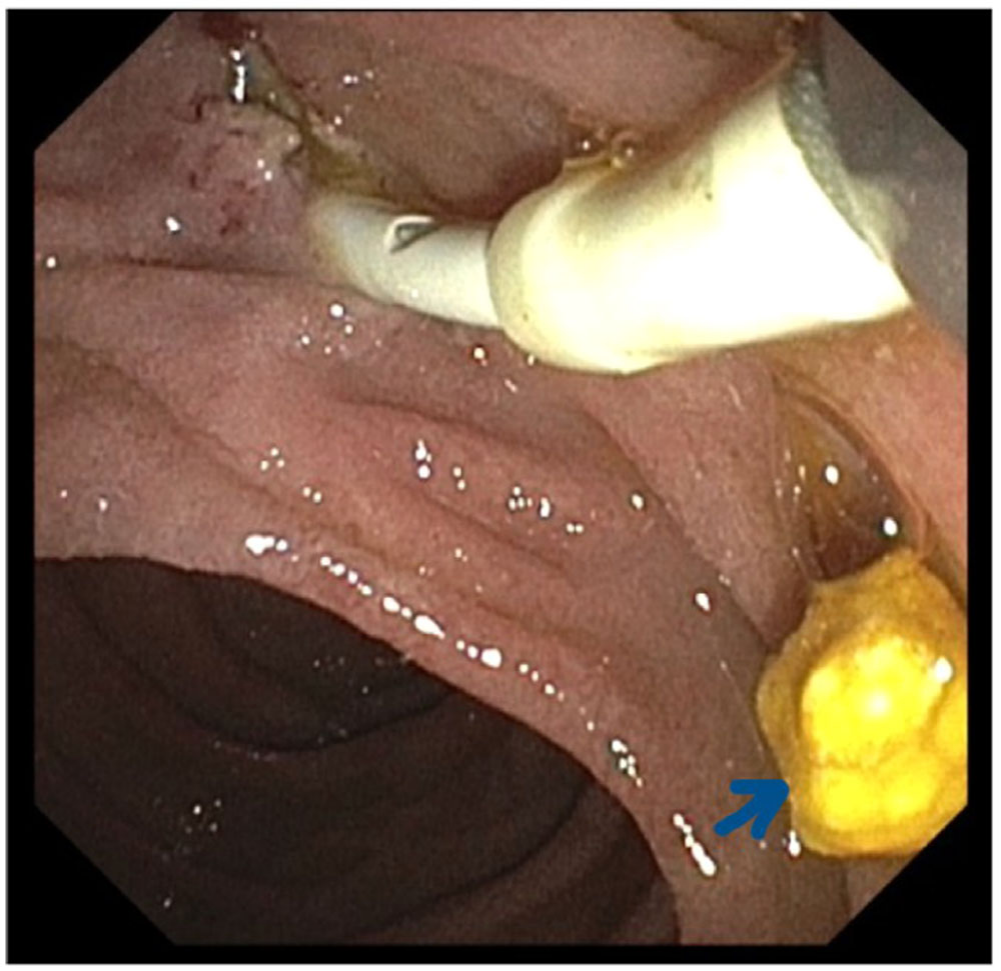
ERCP image showing sphincterotomy and extracted gallstone (arrow). ERCP, Endoscopic retrograde cholangiopancreatography.

She was admitted for management of symptomatic cholelithiasis. Magnetic resonanace cholangiopancreatography on the following day showed cholelithiasis and choledocholithiasis with the CBD dilated to 10 mm and intrahepatic ducts to 8 mm. On Day 3, ERCP with biliary sphincterotomy retrieved a single CBD stone (Figures 1 and 2). She developed pancreatitis that night, marked by severe abdominal pain and elevated lipase (8016 U/L, normal <160 U/L). She was kept NPO and intravenous fluids given. Her Cr increased to 1.5 mg/dL (normal 0.5–1.0 mg/dL) without oliguria. Elevated lactate dehydrogenase (852 U/L) and reticulocyte count (6.61%), schistocytes on a blood smear, raised concerns for TMA—thrombotic thrombocytopenic purpura (TTP) versus HUS.

Negative stool tests for Shiga toxin made typical HUS unlikely. By Day 6, she developed hypertension requiring Amlodipine. Her labs worsened to hemoglobin (Hb) of 5.6 g/dL, platelets of 11 k/mm^3^, and Cr of 2.45 mg/dL. She received packed red blood cells transfusion for severe anemia. With worsening labs in the setting for TMA, the decision was made to initiate therapeutic plasmapheresis, while awaiting ADAMST13 activity level. After five plasmapheresis sessions, her Hb and platelets improved. However, her Cr peaked at 4.5 mg/dL. ADAMSTS13 activity was high suggesting against TTP. She was diagnosed with non‐Shiga toxin‐related HUS and treatment with a complement inhibitor, Eculizumab, was started following which her renal function began to improve. Renal biopsy later confirmed acute TMA with no signs of chronic injury.

She was discharged home with down‐trending Cr after two doses of Eculizumab. The genetic panel for aHUS was negative indicating secondary aHUS. She continued treatment with Eculizumab and later transitioned to a longer‐acting complement inhibitor (Ravulizumab) to receive a total of 6 months of treatment. Now, 9 months posttreatment, she has normal renal function and no signs of hemolysis.

## 3 DISCUSSION

ERCP was introduced in 1968 as a tool for diagnosing and managing pancreaticobiliary diseases. With advancements in other imaging modalities, ERCP is almost exclusively used for therapeutic purposes. Although ERCP is generally considered safe, it is not without complications such as infections (cholangitis, septicemia), bleeding (immediate or delayed), perforation (pancreatic duct, bile duct, or duodenum), pancreatitis, and biliary stricture.^1^

PEP is defined as a new or worsening abdominal pain within 24 h of ERCP, an elevated serum pancreatic enzyme of more than three times the normal and needing at least 2 additional days of hospitalization.^2^ Risk factors for the development of PEP can be patient‐related (females, younger age, sphincter of Oddi dysfunction) and procedure‐related. A recent study showed that PEP occurred in 5%–20% of pediatric patients. High ASGE scores, pancreatic indications, native major papilla, and cannulation of the pancreatic duct had significant associations. Rectal indomethacin use in adults showed benefits in preventing PEP. However, similar benefits in children are yet to be established. The study noted that the non‐PEP group had a significantly higher proportion of PRSS1 gene mutations, but there was no significant association between PEP and other genetic mutations like SPINK1, CFTR, or CTRC.^3^ Although a few adult case reports are found in literature with TTP‐HUS developing as a consequence of PEP,^4^ this is one of the few pediatric cases reported.

TMA is a disease process characterized by capillary endothelial damage leading to hemolytic anemia, thrombocytopenia, and organ damage. TMA can affect multiple systems, including the kidneys, gastrointestinal tract, nervous system, heart, lungs, and peripheral vasculature.^5^ TTP is caused by ADAMST13 enzyme deficiency due to genetic causes or acquired antibodies. HUS results from complement pathway dysregulation. Typical HUS occurs due to Shiga toxin‐producing infections, while atypical HUS (aHUS) can be primary (genetic defects) or secondary (related to nonenteric infections, medications, malignancy, transplantation, pregnancy, antiphospholipid syndrome, scleroderma, and lupus). The incidence of aHUS is estimated to be two cases/million in the United States.^5^ The pathophysiology behind the development of HUS as a complication of PEP is not fully understood but it is proposed that endothelial damage from inflammatory mediators (such as tumor necrosis factor‐alpha, interleukin‐1 [IL‐1], IL‐6, and IL‐18) released from the inflamed pancreas may trigger this condition.^4^ Another proposed mechanism is that circulating pancreatic proteases activate clotting factors which in turn cause disseminated intravascular coagulation.^6^

Prompt diagnosis and treatment of TMA are crucial, as delays can cause irreversible renal failure potentially leading to death. Supportive therapy remains the cornerstone of treatment and includes maintaining intravascular volume, correcting electrolyte imbalances, and avoiding nephrotoxic drugs. Platelet transfusions are contraindicated unless the patient is actively bleeding, as they can worsen TMA. Plasmapheresis introduced as an effective form of acute management, removes antibodies and other proteins that trigger endothelial damage. However, it does not target the complement pathway which is the underlying cause.^7^ Some recent studies showed that plasmapheresis might not be adequate to induce remission (hematological and/or renal) and it might contribute to progressive renal impairment, in addition to several transfusion‐related adverse effects. Complement 5 (C5) inhibitors have emerged as the primary treatment of aHUS. Eculizumab is a humanized monoclonal immunoglobulin G antibody that blocks the formation of C5b by attaching to C5 and preventing its cleavage. Eculizumab treats acute illness and prevents recurrences. However, the optimal duration of treatment has not been well established.^8^ Another FDA‐approved complement inhibitor, Ravulizumab offers a longer half‐life for dosing every 2 months, unlike weekly dosing with Eculizumab.

The prognosis of secondary aHUS is fair, with about 40% of patients at risk of developing end‐stage renal disease.

Pregnancy‐associated aHUS (P‐aHUS) was considered in the differential diagnosis, but since the patient is beyond the 3‐month postpartum period and lacks potential triggers like maternal‐fetal hemorrhage, infections, pre‐eclampsia, or drug exposure, P‐aHUS was considered unlikely.

We intend to bring awareness among the readers about this rare complication that can lead to devastating patient outcomes without early recognition and appropriate management.

## CONFLICT OF INTEREST STATEMENT

The authors declare no conflicts of interest.

## ETHICS STATEMENT

Informed consent was obtained from the patient's mother to submit this case report.
